# Impact of a disability-targeted livelihoods programme in Bangladesh: study protocol for a cluster randomised controlled trial of STAR+

**DOI:** 10.1186/s13063-022-06987-2

**Published:** 2022-12-17

**Authors:** Lena Morgon Banks, Narayan Das, Calum Davey, Afsana Adiba, M. Mahzuz Ali, Tom Shakespeare, Coral Fleming, Hannah Kuper

**Affiliations:** 1 International Centre for Evidence in Disability, London School of Hygiene & Tropical Medicine, London, UK; 2 BRAC Institute of Governance and Development, BRAC University, Dhaka, Bangladesh; 3 Centre for Evaluation, London School of Hygiene & Tropical Medicine, London, UK; 4 BRAC Bangladesh, Dhaka, Bangladesh; 5 BRAC UK, London, UK

**Keywords:** Disability, Livelihoods, Randomised control trial, Bangladesh

## Abstract

**Introduction:**

There is little evidence on the impact of livelihood interventions amongst people with disabilities. Effective programmes are critical for reducing the heightened risk of poverty and unemployment facing persons with disabilities. STAR+ is a skills development and job placement programme targeted to out-of-school youth with disabilities (ages 14–35) living in poverty. It is a disability-targeted adaptation to an existing, effective intervention (STAR), which has been designed to address barriers to decent work for people with disabilities. This protocol describes the design of a cluster randomised controlled trial of STAR+ in 39 of the 64 districts of Bangladesh.

**Methods:**

BRAC has identified 1500 youth with disabilities eligible for STAR+ across its 91 branch offices (typically a geographical areas covering about 8 km radius from local BRAC office) catchment areas (clusters). BRAC has limited funding to deliver STAR+ and so 45 of the 91 branches have been randomly allocated to implement STAR+ (intervention arm). The remaining 46 branches will not deliver STAR+ at this time (control arm). Participants in the control-arm will receive usual care, meaning they are free to enrol in any other livelihood programmes run by BRAC or other organisations including standard STAR (being run in 15 control branches). The cRCT will assess the impact of STAR+ after 12 months on employment status and earnings (primary outcomes), as well as poverty, participation and quality of life (secondary outcomes). Analysis will be through intention-to-treat, with a random mixed effect at cluster level to account for the clustered design. Complementary qualitative research with participants will be conducted to triangulate findings of the cRCT, and a process evaluation will assess implementation fidelity, mechanisms of impact and the role of contextual factors in shaping variations in outcomes.

**Discussion:**

This trial will provide evidence on the impact of a large-scale, disability-targeted intervention. Knowledge on the effectiveness of programmes is critical for informing policy and programming to address poverty and marginalisation amongst this group. Currently, there is little robust data on the effectiveness of livelihood programmes amongst people with disabilities, and so this trial will fill an important evidence gap.

**Trial registration:**

This study has been registered with the Registry for International Development Impact Evaluations, (RIDIE Study ID: 6238114b481ad) on February 25, 2022, and the ISRCTN Registry (ID: ISRCTN15742977).

**Supplementary Information:**

The online version contains supplementary material available at 10.1186/s13063-022-06987-2.

## Introduction

### Background and rationale

It is estimated that 15% of the global population is living with a disability [1]. In Article 1 of the United Nations Convention on the Rights of Persons with Disabilities (UNCRPD), people with disabilities are considered to include “ … those who have long-term physical, mental, intellectual or sensory impairments which in interaction with various barriers may hinder their full and effective participation in society on an equal basis with others” [2]. The UNCRPD has been ratified by 183 countries and the European Union and represents a legal framework for protecting the rights of people with disabilities and a multisectoral approach to disability inclusion [3].

People with disabilities and their households face a heightened risk of poverty [4, 5]. A major contributor to poverty amongst people with disabilities is exclusion from decent work [6]. For example, people with disabilities were significantly less likely to be employed compared to people without disabilities in an analysis from 15 low- and middle-income countries (LMICs) [6]. Unemployment rates are typically higher amongst women as compared to men, due to the double discrimination from gender norms in many contexts [1]. When people with disabilities are engaged in employment, they are more likely than people without disabilities to be self-employed, work in the informal sector and have lower earnings and less stable employment [1, 7, 8]. Excluding people with disabilities from the labour market has many costs, to both the individual, their households as well as society more broadly [9].

Ensuring “decent work for all” is a core aim of the 2030 Sustainable Development Agenda, specifically in Sustainable Development Goal (SDG) 8 [10]. SDG 8 highlights the importance of disability inclusion within activities designed to promote progress towards the achievement of this Goal, as several of its indicators explicitly call for disaggregation by disability [11]. Furthermore, ensuring equal access to work and employment is codified within Article 27 of the UNCRPD, which has been ratified by 183 countries and the European Union [2]. Still, people with disabilities continue to face multiple barriers to participating in work and developing stronger livelihoods, including discrimination and negative attitudes, poor infrastructural and communication accessibility, failure to provide workplace accommodations, and insufficient skills and qualifications due to earlier exclusion from education [12–14].

There is insufficient evidence on strategies to promote access to decent work for people with disabilities in LMICs [15]. A Rapid Evidence Assessment found only 10 studies that measured the effectiveness of livelihood interventions amongst people with disabilities in LMICs, almost all of which were deemed to have a high risk of bias [15]. Furthermore, qualitative studies have indicated that some livelihood programmes, even disability-targeted ones, are poorly designed and may not result in the development of competitive skills or employment opportunities for participants with disabilities, leading to opportunity and actual costs [12, 13]. Consequently, gathering robust evidence on the impact of livelihood programmes amongst people with disabilities is critical to inform policy and planning.

The proposed study is a cluster randomised controlled trial (cRCT) of the STAR+ programme in Bangladesh. STAR+ is a skills development and job placement programme targeted to out-of-school youth with disabilities living in poverty, which is being run by the non-governmental organisation (NGO) BRAC and other partners in multiple districts of Bangladesh. The STAR+ programme is an adaptation to an existing livelihoods programme (STAR), which has been delivered to over 60,452 youth, including 6973 people with disabilities (11% of total graduates) as of May 2021. A previous RCT of STAR amongst the general population in Bangladesh found that key components of the programme increase labour market participation by 16 percentage points and earnings by 23% [16]. These effects were particularly pronounced amongst women. However, programme implementers have acknowledged that STAR required further adaptations to better support the inclusion of people with disabilities, as people with disabilities have been over-represented amongst the programme’s dropouts and typically only people with mild impairments have been included. Consequently, STAR+ was developed to address the gaps of STAR to improve employment opportunities amongst youth with disabilities.

### Aim and objectives

The overall aim of this research is to assess the impact of the STAR+ programme in improving livelihoods and well-being amongst youth with disabilities in Bangladesh.

Specific objectives include:

1. To evaluate the impact of STAR+ on employment and earnings amongst youth with disabilities (primary outcomes).
2. To estimate the impact of STAR+ on poverty, participation and quality of life amongst youth with disabilities (secondary outcomes).
3. To assess differences in impact amongst youth with disabilities (e.g. by gender, impairment type)
4. To explore what aspects of the STAR+ programme were perceived to be most important for affecting desired impacts amongst youth with disabilities
5. To examine challenges and enablers to the implementation and delivery of STAR+ from the perspective of participants and implementers

## Methods and analysis

### Design

The study’s main design is a multicentre, superiority cRCT. BRAC’s 91 branch office catchment areas will serve as the clusters for this trial. These clusters cover 39 of the 64 districts of Bangladesh. Of the 91 branches, 45 have been randomly allocated to being implementers of STAR+. The remainder of the branches will serve as control areas and will not implement STAR+ during the study timeframe. Control areas may implement other livelihood programmes (e.g. 15 were randomly allocated to implement standard STAR). BRAC has identified youth with disabilities meeting the STAR+ eligibility criteria in both the control and intervention clusters. Baseline data collection was collected after randomisation but before participants were invited to enrol in STAR+ to minimise anticipatory behaviour. Follow-up will be conducted 17 months after the completion of the delivery of STAR+ (24 months after baseline).

The cRCT will be complemented with qualitative research and a process evaluation. For the qualitative, in-depth interviews will be conducted with participants in both study arms at baseline and endline to explore their experiences of employment, and for the intervention arm, of the STAR+ programme. For the process evaluation, in-depth interviews with programme implementers will be conducted and monitoring data from BRAC and the endline of the cRCT will be reviewed to explore the implementation fidelity, mechanisms of impact, and contextual factors that can affect variations in outcomes. It will use the Medical Research Council guidance for process evaluations of complex interventions as a framework [17]. Researchers from the International Centre for Evidence in Disability at the London School of Hygiene & Tropical Medicine in the UK and the BRAC Institute of Governance and Development in Bangladesh will jointly design and implement the research.

### Eligibility criteria

Participants in both the control and intervention arms must meet BRAC’s eligibility criteria for STAR+. Eligibility for STAR+ is based on the following conditions: (a) having a disability; (b) age is between 14 and 35 years; (c) dropped out of school for at least a year, (d) not currently in employment or training; and (e) currently living in poverty, meaning their household earns BDT 4000 (US$46) or less per capita per month.

Determining disability is methodologically complex, particularly for deciding programme eligibility [18, 19]. For STAR+, BRAC considers youth to have an eligible disability if they either have a disability identification card issued by the national government, or if they were identified in a door-to-door survey conducted in consultation with local Organisations of Persons with Disabilities (OPDs). Permanent residents can apply for disability identification cards, which involves a medical assessment to determine if they have a disability in line with the Persons with Disabilities Rights and Protection Act (2013) [20]. This legislation defines disability as including physical, sensory, intellectual, communication and psychosocial impairments, including specific conditions such as autism and Downs Syndrome [20]. The criteria used in the survey are aligned with UNCRPD and the national definitions of disability [21]. People with deafblindness and more severe intellectual impairments will not be part of the cRCT, as they will be included in specialised STAR+ pilot programmes that will be evaluated separately. All identified individuals will also be asked the Washington Group Short Set of questions [22]. These questions are widely used and the United Nations’ recommended questions for measuring disability prevalence in surveys to allow for international comparisons [23].

### Recruitment and randomisation

Across all clusters, 1500 youth with disabilities who are eligible for STAR+ were identified by BRAC through a door-to-door survey conducted with assistance from OPDs in December 2021. Branch office catchment areas (clusters) were then randomly allocated using random number generated by Stata by the research team at the London School of Hygiene & Tropical Medicine (LSHTM) within divisions (administrative unit above district). The allocation decisions were then provided to the programme implementer, BRAC, to inform which of their clusters (branch offices) would be implementing STAR+, and therefore which identified individuals would be invited to enrol. Clusters in the control arm will not run STAR+ during the duration of the trial. It is expected that cluster randomisation will achieve a roughly 1:1 allocation of participants (750 in each arm). Randomisation occurred after identification but before the enrolment of participants into STAR+, meaning participants, data collectors and programme implementers were unaware of allocation decisions at the time of baseline data collection.

Cluster-randomisation is appropriate because of the community-level delivery of many of the project components (e.g. community sensitisation events). Cluster-level variation in receipt of the intervention may reduce the potential for resentment, as participants will not be masked from allocation. Furthermore, cluster randomisation can help to fairly allocate an intervention that cannot be delivered to all eligible people.

For the qualitative research, 15–20 participants will be recruited from the cRCT at endline. Recruitment will be purposive to maximise heterogeneity by gender, impairment type and study location. For the process evaluation, 15-20 implementers of STAR+ will be interviewed after the completion of STAR+. Implementers will be selected to reflect diversity of roles and will include BRAC staff who helped develop and carry out the STAR+ programme, as well as other partners who were key to its delivery (i.e. employers, instructors, OPDs involved in selection).

### Intervention

Youth with disabilities in the intervention arm will be invited to enrol in STAR+, which will be delivered in all of the intervention areas alongside the conventional STAR programme. The STAR+ programme was developed by BRAC and partners through consultations with key stakeholders (youth with disabilities, OPDs, government actors), a context analysis and needs assessment, which were conducted between October 2018 and April 2019. This formative research highlighted key barriers to decent employment faced by youth with disabilities in Bangladesh to inform the design of the STAR+ programme.

STAR+ will be delivered over 7 months, with an additional 3 months for preparation (i.e. identification of participants). The core components of STAR+ in the 7 months of implementation include:

Months 1–6

1. Sensitisation events with families, communities and employers: Community events will address stigma, discrimination and misconceptions about the ability of people with disabilities to work. These events will use behaviour change messaging that has been developed by BRAC through formative research.
2. Provision of assistive devices and rehabilitation support: Participants will undergo a medical assessment during enrolment, and those who are assessed as having unmet needs for assistive devices or rehabilitation support will be provided with them by BRAC.
3. Accessible and inclusive technical and soft skills training: Participants will choose a preferred trade from a list that has been developed based on local labour market consultations. Over 6 months, participants will receive on-the-job training in their chosen trade five days a week, and classroom training for 1 day a week. Adaptations have been made to the trainings to ensure they are accessible and inclusive of people with disabilities. For example, instructors and master tradespeople involved in both the classroom and on-the-job trainings will receive their own training on disability inclusion and inclusive facilitation. Additionally, workplaces for the on-the-job training placements will undergo accessibility audits and BRAC will monitor the employers to ensure decent work conditions. Participants will receive a stipend from BRAC during their training.

Month 7

(4) Job matching: Near the end of the 6-month training, BRAC staff and partners will identify waged job placements for participants in their chosen trade. It is expected that these placements will primarily be with enrolees’ current training workplaces, although other employers will be identified for individuals who are not retained. BRAC staff will monitor the transition to paid work for three months. There is no set length to the job placements.

### Usual care

Youth with disabilities in the control arm will receive usual care, meaning they will not be offered enrolment in STAR+ for the duration of the trial. However, they are free to access any services or programmes operating in their area. In a minority of clusters, BRAC (n=14) will be offering the standard STAR programme. Recruitment for the standard STAR – and any other livelihood programmes delivered by BRAC or other organisations – will continue as usual, with control-arm participants neither purposively included nor excluded. Control-arm participants will be provided with information about available social protection programmes in Bangladesh during baseline. Data on enrolment in other programmes will be captured during the endline survey.

### Data collection

Data for the cRCT will be collected using questionnaires completed by the 1500 participants in the intervention and control clusters at baseline and endline. Other household members may answer some sections of the questionnaire, for example, sections on household finances or membership. Participants in the control and intervention arms identified by BRAC will be visited in their homes by the research team. The questionnaire is based upon standard modules previously used in the original STAR evaluation [16] and from modules used in other studies [24–26] (Table 1). It includes sections on household composition and demographics; employment, earnings, credits and savings of all household members; household expenditures; individual well-being and social participation. It was piloted before full-scale data collection to check for acceptability and understanding. The survey will take approximately 1 hour to complete and will be delivered by trained enumerators using SurveyCTO.

**Table 1:** Outcome indicators for the cRCT of STAR+

Outcome indicator	Description	Source
*Primary outcomes*		
Employment	Engaging in any activity for pay or profit (including in kind) during the preceding month.	SDG indicator 8.5.1 [27]
Earnings	Average hourly and total earnings in the preceding month. Market equivalent cash value will be used to estimate in-kind payment values.	SDG indicator 8.5.2 [27]
*Secondary outcomes*		
Monetary poverty	Household consumption and income per capita is below national poverty line	SDG indicator 1.2.1 [27]
Multidimensional poverty	Household defined as poor using an adapted version of the Global Multidimensional Poverty Index (2020 revision) [28]	SDG indicator 1.2.1 [27]
Subjective wellbeing	Total score on an 8-item tool on self-reported well-being	Wellbeing of Older People Study [24]
Social attitudes	Total score on an 9-item tool on social attitudes	World Health Model Disability Survey [25]
Empowerment	Total score on an 7-item tool on decision-making	RCT of STAR [16]
Violence	Experienced violence (physical, verbal) or discrimination in the last 12 months	SINTEF Living Conditions Surveys [26]

In-depth interviews will be conducted with 15-20 participants of the cRCT at endline, and with 15-20 programme implementers as part of the process evaluation. Interviews will use semi-structured topic guides and will be conducted by experienced researchers. They will be recorded, transcribed and translated to English. Interviews with cRCT participants will focus on barriers and enablers to seeking and retaining work and experiences on the job. STAR+ enrolees will also be asked about the perceived impact (if any) of the programme, the strengths and areas for improvement in different components to the programme, and any recommendations for adapting the overall programme. Interviews with implementers will focus on their experience developing and/or delivering the components of STAR+ they were responsible for, including any challenges, adaptations and suggestions for improvements.

Participants will be asked for multiple modes of contact during baseline to increase the response rate at endline.

### Outcomes

A theory of change was developed with input from BRAC to identify the anticipated outcomes of STAR+. The ultimate aim of STAR+ is to improve opportunities for decent work amongst youth with disabilities, which can lead to reduced poverty and improved participation and well-being. The primary outcome measures are employment and earnings (Table 1). Secondary outcome measures will include household poverty and the participant’s subjective well-being, social participation/empowerment and experiences of stigma and discrimination. Indicators are tied to the SDGs where possible.

Furthermore, the endline survey will collect information from the intervention arm about their experience participating in the programme, including (a) details on receipt of different components of the intervention to explore fidelity and uptake (e.g. what was received, frequency, how delivered); (b) satisfaction with the programme overall and with specific components; (c) challenges experienced during any component of the intervention; and (d) self-reported impacts of participation.

### Sample size

Sample size calculations were conducted in R using the ‘clusterPower’ package. The programme will work with approximately 750 learners in 45 of the 91 eligible BRAC branch offices; we have therefore assumed that each branch could work with 16 learners (1500/91). Sample size calculations are based on the primary outcome of the proportion of learners in employment. The possible effect size was calculated using the proportion in employment in the STAR evaluation (53%) and the proportion in the control (41%). For the proportion in the control, an ‘exclusion factor’ of between 0 and 50% was applied to reflect the various probabilities that the employment amongst those eligible for STAR+ will be lower than what was observed amongst STAR participants. As the level of clustering is unknown, the study power was estimated for a range of values.

**Fig. 1:**
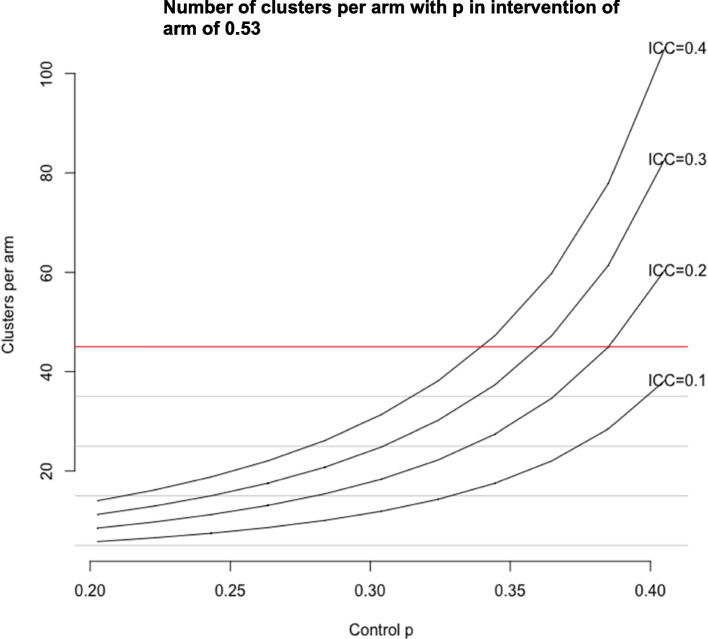
Sample size calculation

The results of the sample size calculations are shown in Fig. 1. Each line shows the number of clusters required in each arm to achieve 80% power for different levels of employment in the control arm and for different intraclass correlation coefficients (ICCs). When the ICC is low (0.1), the trial will have sufficient power for up to and including a control-arm proportion equal to that observed in the STAR trial. For a more conservative ICC of 0.2, the trial will be sufficiently powered when the control arm has 95% of the employment seen in STAR. As the ICC increases, the power is reduced, however with an ICC of 0.4 — representing high levels of clustering — the trial will still be sufficiently powered so long as the employment in the controls is approximately 75% of that observed in STAR. Since we expect that there is a substantial effect of disability on employment in this context, we consider this to be adequately powered.

### Timeline

Baseline data collection is being conducted January-February 2022, in sync with recruitment. The process evaluation will be conducted at the end of STAR+ delivery. Endline data collection will take place approximately 17 months after the completion of STAR+ (approximately 24 months from baseline) to control for seasonal variations that can affect outcome measures.

### Data analysis

A detailed analysis plan will be published before the endline survey. The analysis will estimate intention-to-treat (ITT) effects of STAR+.

The quality of the balance achieved by randomisation will be assessed by describing the arms of the trial at baseline in terms of the primary and secondary outcomes and sociodemographic variables. If there is evidence of imbalance, based on subjective interpretation of the magnitude of the difference, such variables will be included *a priori* in the main analysis of the primary and secondary outcomes.

The effect of the intervention will be estimated by comparing the proportions (e.g. employment) and the means (e.g. earnings) between the arms of the trial. The unadjusted estimated effects will be reported as risk ratios for binary outcomes and difference in the means for continuous outcomes. In the final analysis, to increase the precision of the estimates and reduce the risk of bias from imbalances at baseline, regression will be used to adjust for the baseline levels of the outcome, stratification variables, and variables considered to be imbalanced at baseline. For binary outcomes, the risk ratio will be modelled with a modified Poisson regression model [29]. Linear regression will be used for continuous outcomes. A random mixed effect at the cluster level will be used to account for the clustered design.

In-depth interviews will be recorded, transcribed and translated. Detailed notes and transcripts will be analysed using thematic analysis. Coding frameworks will be developed using the semi-structured interview guides as a starting point. Additional codes emerging from the data will be incorporated into the framework in an iterative process. Transcripts and notes will be coded using NVivo 12. Codes will be grouped into themes and sub-themes. Comparisons and inter-relationships between themes and sub-groups (e.g. women vs men, by impairment type) will be conducted throughout the analysis. The process evaluation will also use data from the endline cRCT and monitoring data from BRAC on the receipt and user experience of different components of STAR+. This data will be tabulated and regression used to explore differences amongst STAR+ participants (e.g. by gender, location, impairment type).

### Ethics and dissemination

Ethical approval has been received from the institutional review boards at the London School of Hygiene and Tropical Medicine (UK) and the BRAC Institute of Governance and Development (Bangladesh).

Written consent will be sought from all participants by trained data collectors (Supplementary File 1). Bangladesh does not have a national age of consent. As such, direct consent will be sought for all individuals aged 18 and older and participants ages 14–17 if they are married or working. Parent/guardian consent will be sought for participants 14–17 years who are neither married nor employed, and participants will provide their assent. Capacity to consent will be assessed through an “Evaluation to Sign Consent” [30], which asks participants five questions about their understanding of the information sheet. Participants who are unable to provide a satisfactory response even with clarifications will be deemed unable to provide true informed consent. In these instances, parents/guardians will provide their consent and the participant will provide assent. Adaptations will be in place to support the direct participation of people with different impairments (e.g. sign language interpretation, simplified interview schedules and information sheets).

Data from participants in the cRCT and qualitative research will be fully anonymised. Participants in the process evaluation will be advised during the informed consent process that they may be identified by job title unless requested otherwise. Data storage and management protocols are governed by a Data Protection Impact Assessment. Anonymised data will be made available on the LSHTM Data Compass [31]. Study findings will be disseminated widely, including through webinars, peer-reviewed journal articles and short reports.

No specific discomfort, distress or hazards are expected as a result of any component of the research (participating in survey, in-depth interviews). Participants may feel uncomfortable discussing their experiences, but will be reminded that they have the right to stop or refuse to answer any questions at any time, for any reason. Precautions will be taken in light of the ongoing COVID-19 pandemic, including following national guidelines on in-person meetings, provision of personal protective equipment to research teams, and giving participants options to participate remotely. Participants will not be compensated for taking part in the research activities, but STAR+ enrolees will be provided a stipend by BRAC during their training and then will be paid wages commensurate with their role in their job placement. BRAC has in place separate monitoring procedures to report and address any harms arising from participation in the programme.

A concern for a study of this nature is fulfilling equipoise, as it would not be ethical to evaluate an intervention such as STAR+ if there was a certainty that it would be more beneficial than the usual care alternative provided to the control group. However, past qualitative research in Bangladesh and other settings has highlighted that trainings and skills development programmes, including disability-targeted programmes, may provide little benefit for people with disabilities [12, 13]. For example, participants in different livelihood programmes have reported that they felt they did not gain practical skills that were suitable for their local job market, or were diverted from continuing to develop a pre-existing trade/skill set. In worst case scenarios, improperly implemented livelihoods programmes have caused harm: for example, an asset transfer programme in Honduras resulted in worsening socioeconomic status as households invested time and money into their assets, which in turn did not lead to income generation [32]. Participating in these programmes therefore carries at a minimum opportunity and potentially actual costs (e.g. travel to training/workplace), and it is unknown the extent to which these costs outweigh the benefits of participation. Further, BRAC has limited funding to implement STAR+ and cannot deliver it at this time to all people who have been identified, and so random allocation represents an equitable division of scarce resources.

LSHTM takes primary responsibility for the design of the study and ensuring it meets appropriate standards. Any concerns or instances of misconduct related to participating in the research (i.e. survey, in-depth interview) can be reported to the LSHTM Research Governance and Integrity Office (rgio@lshtm.ac.uk), which is separate from the research team. BRAC is responsible for the delivery of the intervention, and for monitoring and addressing any harms that result from participation in the intervention.

The study funder (United Kingdom Foreign, Commonwealth and Development Office) and programme implementer (BRAC) will not be involved in data collection or management, analysis or publication decisions. The programme implementer was consulted during the study design, to ensure that trial outcomes were in-line with the intended outcomes of the programme. Authorship on any papers or reports will be determined according to standard guidance [33]. Any important protocol modifications will be updated within the trial registry.

## Discussion

This study on the impact of STAR+ will be one of the few trials of a livelihood intervention amongst people with disabilities [15].

STAR+ is an important programme to evaluate for several reasons. First, it is a large-scale intervention, which is being delivered across 39 of the 64 districts of Bangladesh. Evidence on its effectiveness and on what, if any, adaptations are required for further improvements can help justify continued investment and improve STAR+ design and delivery. Second, STAR+ has been adapted from an existing programme (STAR), which has been implemented widely in Bangladesh. A RCT of STAR found that it improved livelihood outcomes such as employment status and earnings amongst the general population, particularly in women [16]. STAR+ therefore has a strong foundation underscoring its approach. Third, the process for adapting STAR to the disability-targeted STAR+ involved extensive consultations and formative research, including the active involvement of youth with disabilities and OPDs. This method for project design is evidence-based and in line with the principles of the UNCRPD. Investigating the effectiveness of a programme designed in this way, and exploring in detail the implementation of this approach through a process evaluation, can provide evidence on disability-inclusive programme design. Finally, STAR+ programme intends to reach a broad range of participants, including people with different impairment types and in different settings (e.g. rural/urban, dominant local industries) across Bangladesh. This reach improves generalisability of the trial’s findings.

There are some limitations to this study design. Importantly, blinding was not possible in a study of this nature, which could introduce bias. However, most outcome measures (e.g. employment status, earnings, household income) are objective in nature. Consequently, the risk of participants or data collectors reporting outcomes differently between trial arms is considered low. Additionally, some people with disabilities may not be reached through the form of STAR+ being evaluated. For example, people with deafblindness and severe intellectual impairments in the intervention areas are not included in the main STAR+ that is being evaluated in this study. Instead, separate pilot programmes are being run for these groups. The enrolment numbers in these pilot programmes are inadequate for evaluation within the RCT, although other qualitative research is planned by other groups. As such, the study findings are unlikely to be generalisable to these groups.

## Conclusion and impact

In summary, this research is significant because it (1) provides one of the few trials of a livelihood intervention amongst people with disabilities in a LMIC setting and (2) evaluates a large-scale, wide-reaching, disability-targeted programme that has been adapted from an effective non-targeted intervention through collaboration with people with disabilities and formative research. Findings from this research have the potential to inform the design and delivery of not just STAR+, but other disability-inclusive livelihoods programmes.

Governments, NGOs and other organisations have limited resources to provide interventions such as the STAR+ programme, particularly in light of budget cuts to foreign aid due to COVID-19. The dearth of evidence on the effectiveness of livelihood interventions amongst people with disabilities impedes informed policymaking and planning. Data on the effectiveness of STAR+ can therefore build a case for continued investment in the programme — including the potential adaptation and expansion to other contexts — and/or indicate areas through which to improve the programme.

## Trial status

This protocol is version 3 (January 4, 2022). Participant recruitment began in January 2022 and ended in February 2022.

## Supplementary Information

**Additional file 1.**

## Data Availability

An anonymised version of the trial dataset will be available upon request from the London School of Hygiene & Tropical Medicine’s Data Compass.
