# Supplementary material for: Impact of a disability-targeted livelihoods programme in Bangladesh: study protocol for a cluster randomised controlled trial of STAR+
Source: Trials. 2022 Dec 17;23:1022. doi: 10.1186/s13063-022-06987-2 (PMC9758025; doi:10.1186/s13063-022-06987-2)
Supplement: Supplementary file 1 — Additional file 1. [file 13063_2022_6987_MOESM1_ESM.docx]

**September 30^th^, 2021**

**Participant Information Sheet – Qualitative research**

**Title of Project:**  *Impact evaluation of the STAR+ skills development programme in Bangladesh*

**Introduction**

We would like to invite you to take part in a research study. Joining the study is entirely up to you. Before you decide, you need to understand why the research is being done and what it would involve. I will read this information sheet to you now. Ask questions if anything is not clear or you would like more information.

**What is the purpose of the study?**

The London School of Hygiene & Tropical Medicine (LSHTM), a university in the United Kingdom, and the BRAC Institute of Governance and Development (BIGD), a research institute in Bangladesh, are conducting research about people with disabilities and work. This includes their experiences of finding and being at work, as well as reasons they may or may not be working. This research also is looking at the impact of different programmes that can help people with disabilities in finding and staying in work.

**Why have I been asked to take part?**

You have been invited because our records indicate that you have a disability or a health condition. You may have been identified as a possible participant by either an organisation working in this area or because you receive a Golden Citizenship card from the government.

**Do I have to take part?**

No. It is up to you to decide to take part or not. Your decision to participate or not participate will have no effect on any services you receive now or in the future. We will not share your decision with anyone outside the research team, including the organisation that identified you as a possible participant.

**What will I have to do?**

If you agree to take part, I will first ask you to [*delete as needed*: sign/thumbprint a document confirming you’ve been told about the study and that you agree to participate OR confirm you’ve been told about the study and that you agree to participate by being recorded agreeing to the study terms].

We will then have a discussion where I will ask you some questions about your work experiences (including reasons why you might not be working). I will also ask about your participation in employment and training programmes. The discussion will last about 30 minutes and 60 minutes.

We will record the interview so that I can accurately remember what you have told me. You can end this interview at any time or refuse to answer any of my questions.

We would also like to talk to you again in about 1-2 years, but you can decide at that time if you would like to talk to us again or not.

**What are the possible risks and disadvantages?**

There are no risks to participating, although it is possible you may feel distressed at discussing your experiences.

**What are the possible benefits?**

Participation in this study is completely voluntary. We are unfortunately not able to provide any cash or other benefits for your participation. The information we get from the study will help our knowledge and understanding of how to improve employment opportunities for people with disabilities.

**Who can I contact for more information?**

If you have a concern or want more information about any aspect of this study, you can ask me any questions. You can also speak to other members of the researcher team who will do their best to answer your questions (contact details below).

**Can I change my mind about taking part?**

Yes. You can stop the discussion at any time without giving a reason. There will be no negative consequences for you if you decide to withdraw from the study.

**What will happen to information collected about me?**

All information collected about you will be kept private. A member of the research team will listen to the recording and write up everything we say on the recording. I will also take notes of our discussion today. The write-up of the discussion and my notes – from you and other people - will be used as data in this research study.

Your data will be anonymised. This means that we will remove personal details, like your name, contact details and address, so that you cannot be recognised when looking at your data. Only the study staff and authorities who check that the study is being carried out properly will be allowed to look at your personal details, like your name and contact information. Your personal details will be kept in a different safe place to the other study data (e.g. recordings, write-up, notes), and will be destroyed within 10 years of the end of the study. A copy of your data will be sent to the research team at LSHTM in the United Kingdom, but this will be anonymised.

We may include details that you share with us on an online data repository. A data repository is a site where other researchers outside this study team can look at data from different studies so that they can confirm our findings or use your data for other research purposes. All information that could potentially identify you will be removed before we put it on this site.

**What will happen to the results of this study?**

The study results will be published in a journal so that policy-makers and practitioners can learn from them. We will also share the learning with policy-makers and practitioners directly. Your personal information will not be included in any study materials so that you can’t be identified from it.

For more details, contact:

XXXX

BRAC Institute of Governance and Development

**Impact evaluation of the STAR+ skills development programme in Bangladesh**

# PLEASE INITIAL THE RELEVANT BOXES Initials

1. I confirm that I have read, and that I understand, the Participant Information Sheet. I have had the opportunity to consider the information provided, ask questions about the study, and have had these answered satisfactorily.

1. I understand that my participation is voluntary and that I am free to withdraw from the study at any time, without giving a reason.

1. I consent to the information I provide being stored on the computers at LSHTM and BIGD.
2. I consent to using my anonymised data (i.e. no names, contact details or any other information that can be used to identify me) being made available on an online data storage site that other people can access.
3. I understand that if I inform the researcher that myself or someone else is at risk of harm they may have to report this to the relevant authorities - they will discuss this with me first but may be required to report with or without my permission.

## Signature/Thumbprint of research participant

---------------------------- ----------------------------------------- ----------------

Name Signature of participant Date

## Signature of the witness (needed only if thumbprint signed)

---------------------------- ----------------------------------------- ----------------

Name Signature of witness Date

## Signature of researcher (needed only if thumbprint signed)

I believe the participant is giving informed consent to participate in this study

------------------------------ ------------------------------------------ ----------------------

Name Signature of researcher Date
